# The effect of language on prosocial behaviors in preschool children

**DOI:** 10.1371/journal.pone.0240028

**Published:** 2020-10-06

**Authors:** Eszter Somogyi, Thuy Tuong Uyen Tran, Bahia Guellai, Ildikó Király, Rana Esseily

**Affiliations:** 1 Department of Psychology, University of Portsmouth, Portsmouth, United Kingdom; 2 Department of Psychological Sciences, University Paris Nanterre, Nanterre, France; 3 Hungarian Academy of Sciences - Eötvös Loránd University Social Minds Research Group, Budapest, Hungary; Middlesex University, UNITED KINGDOM

## Abstract

The present study investigated how linguistic group membership influences prosocial behaviors, namely helpfulness and cooperation, in preschool children. Whilst research indicates that children preferentially direct their prosocial behavior towards members of their own groups, the influence of perceived linguistic group membership on actual helpfulness and cooperation has not been investigated. We presented an experimenter to 4- and 5-year-olds either as a foreigner, who did not speak the local language or as a native person. Children were then given the opportunity to help or cooperate with this experimenter in a series of nonverbal playful tasks. Whilst 4-year-olds helped and cooperated equally with the foreign and the native experimenter, 5-year-olds required significantly more cues and prompts in order to help or cooperate in the foreign condition. We also found that children were overall more reluctant to respond prosocially in the cooperation tasks than in the helping tasks. We tested children in two European countries (France and Hungary) and found the same pattern of responses in the two locations, suggesting that our findings are not specific to the local culture. Our results extend the findings of earlier research that showed selectivity according to the language spoken by the partner for sharing and imitation. Studies that looked at helpfulness or cooperation used the minimal group paradigm to induce group membership (based on arbitrary cues) and used indirect measures of prosociality, such as different forms of reasoning about the partner. In our study, we used language, a natural cue for group membership (versus arbitrary cues or cues based on social conventions) and directly observed children’s helpful and cooperative behaviors toward the experimenter. Our results also confirm previous results indicating that with age, children become selective in their prosocial behaviors as they acquire new means of social evaluation and categorization. We conclude that the language associated with a potential social partner is not only a cue for affiliation and shared knowledge but also a cue mediating children’s prosocial acts.

## Introduction

Humans appear exceptional in their ability to respond to the needs of their conspecifics [1], very early in development [2]. Prosocial behavior refers to a wide range of actions intended to benefit or support others, such as helping, cooperating, sharing, comforting or informing. Prosocial behaviors emerge in infancy and develop throughout the preschool years [1, 3, 4]. For instance, young children help others achieve instrumental goals [5], share resources [6], show natural concern when others are hurt [7], and provide information to others [8]. Obeying the rules and conforming to norms are also regarded as prosocial behaviors [3]. Although the prosocial behaviors that children can produce are well documented, we still have much to learn about the factors that modulate them.

In infants and toddlers, spontaneous helping behavior is indiscriminate and intrinsically motivated. By around 14 months, they help unfamiliar adults in both experimental (e.g., [5, 9]) and observational (e.g., [10, 11]) studies. While 3-year-olds share less over time with a consistently selfish partner, 2-year-olds continue to share regardless [12, 13]. Similarly, neither praise nor rewards [14] or the presence and encouragement of caregivers [15] increases toddlers’ helping behavior. Two-year-olds’ physiological arousal, as measured via changes in pupil dilation, is equally reduced when they themselves help or a third party helps someone [16], which also suggests that toddlers are not driven by a possible reward for having themselves provided help.

During their preschool years however, children become more and more selective in their prosocial behaviors, acting more prosocially towards certain partners or groups than others [2]. Some authors argue that this selectivity is in fact already present in infancy [17], guiding infants’ earliest social preferences. Indeed, infants prefer helpful agents over harmful ones (e.g., [18]), or look longer to faces of individuals who are from familiar rather than unfamiliar groups (e.g., [19]). Some existing studies have looked at the role of social factors such as affiliation, trust or group membership in guiding this selectivity. Still, only a limited number of studies have investigated the role of the language spoken by the other person, a powerful cue for these social factors [20]. The current paper therefore seeks to clarify how the status of another person, an adult presented as a native or a foreign individual influences prosocial acts, namely helping and cooperation, in preschool children.

Helping is an altruistic behavior with no direct benefit to oneself and requires the capacity to represent the other person’s unfulfilled goal. Cooperation, on the other hand, is based on the formation of a shared goal and the cooperating partners must mutually support each other’s action to reach that goal. From a developmental perspective, it has been proposed that actively helping others fulfil their goals is an early form of cooperation as it provides the benefit of seeing the need of the partner met and because both originate from an early existing prosocial motivation [16, 21]. In this paper we will examine both helping and cooperation as two forms of early prosocial behavior.

Before presenting our study, we discuss the social factors that have been shown to influence prosociality in preschoolers, such as their need to affiliate with others and their increasing knowledge about their own and other social groups. We also examine the role of the language spoken by potential social partners in mediating these effects.

### Affiliation and trust affect prosocial behaviors

During their preschool years, children show a growing need to affiliate with others and care about the relationship they have with those around them. They are more generous toward their friends and schoolmates than they are toward disliked peers, strangers or children from other schools and this is even more prevalent as they get older [22–24]. Preschoolers share or help more when they feel sympathy or empathy towards the other child [25, 26], or when they are primed with affiliation [27]. They take into account the closeness of the recipient’s relationship to the sharer when guiding a puppet to give out resources to strangers, friends and siblings [28].

In terms of the effects of friendship on helping, one study found that 3-year-olds were not only more likely to help a friend than a neutral peer in a forced-choice setting, but also demonstrated a greater overall motivation to benefit a partner if that partner was a friend, as measured in this case by the amount of paper shreds they helped to clean up [29]. In fact, even before the preschool age, infants have been shown to help more after being mimicked by a friendly adult [30], possibly because mimicking creates a social bond [31].

Beyond affiliation, the partner’s trustworthiness, based on their observed helpfulness [28, 32], their intentions and moral character [33] as well as previous collaborations with the partner [34] also guide preschoolers’ prosocial acts. Do children engage in prosocial behaviors when they have no personal history or relationships with the other person but nonetheless share their membership in a social category?

### Social categorization affects prosocial behaviors

A few studies explored how social categorization based on cues such as race, gender or language influences prosociality in children, focussing mostly on how preschoolers share resources, such as stickers or toys. In a study by Renno and Shutts [35] 3-to 5-year-old Caucasian children were asked to distribute token coins to target children (presented in paired photographs), who varied by either race or gender. Children gave more resources to unfamiliar Caucasian than to unfamiliar Afro-American targets and were also more generous toward unfamiliar same-gender (vs. unfamiliar other-gender) children. To investigate the effect of language, Kinzler, Dupoux and Spelke [36] presented 2.5-year-old children in both the U.S. and France with videos of one person who spoke in English, and another person who spoke in French. In a subsequent game, children could give a ‘present’ to one of the two speakers. The authors found that children reliably gave this to the native speaker belonging to the same linguistic ingroup. To explore the effect of group membership alone, in the absence of any prior experience with in- or outgroup members, Dunham, Baron and Carey [37] used the so-called minimal group paradigm, inducing group membership with arbitrary cues such as hat color, sticker type. Five-year-olds who were thus randomly assigned to one group showed a range of biased behaviors towards their own minimal group: preferring ingroup individuals; making more positive associations and giving somewhat more resources to ingroup members.

To our knowledge, only two studies have looked at the effect of social categorization on helping behavior and these were conducted with slightly older schoolchildren. A study by Katz, Katz and Cohen [38] investigated the effects of ethnic group membership by providing Caucasian children aged 5–10 years the opportunity to help an experimenter, who was either African American or Caucasian, to set up materials for the next participant. Although children helped both experimenters, they helped the Caucasian experimenter for longer and more comprehensively than they did the African American experimenter. Another study by Bigler, Jones and Lobliner [39] used minimal groups, induced with yellow and blue coloured t-shirts, to study 6–9-year-olds’ willingness to help their peers. Whilst children showed ingroup preference overall (making more positive associations and giving somewhat more resources to ingroup members), their actual helping behavior did not depend on the group membership of the receiving peer. In fact, children were largely unwilling to help members of either group, which could equally be explained by their reluctance to help or by the possibility that the task itself was not engaging for this age group (stringing together a pile of plastic squares). It is also possible that minimal cues were insufficient for inducing group membership in this age group.

We can see that most of the existing literature has focussed on the effects of social categorization on sharing. The two studies that looked at helping behaviors in older children show mixed results and have not looked at the effect of the language. This is surprising, given that language is a powerful everyday cue that guides children when choosing who to affiliate with and who to learn from.

### Language as a natural cue for affiliation, learning and prosociality

From birth, infants have a natural preference for their own language [40, 41] and will choose or accept items more readily if they are proposed by persons who speak their native language as compared to when these are offered by a non-native (see [42] for preference of tunes proposed by a native speaker; [36] for toy choice and [43] for food preference).

Language is also an important cue for selective learning from members of our own linguistic group, through imitation for instance. Several studies report that young infants imitate a novel action more often when the model speaks in the infant’s native (vs. a foreign) language [44–46]. Beyond imitation, language conveys the specific rules and conventions that a community share. For instance, 2-year-olds associate a foreign language with a model if he had previously used tools in a non-conventional (vs. conventional) way [47].

Language thus guides children when choosing the person to affiliate with, to trust and to learn from. Earlier, we have seen that affiliation and group membership modulate children’s prosocial behaviors [24–39] and that the status of another person as a native or a foreign individual influences sharing [36, 37]. It seems plausible that linguistic group membership also modulates helping and cooperation [20].

### The present study

We therefore wished to learn more about how the language spoken by another individual influences prosocial behaviors towards this person in young children. We presented a female adult experimenter to 4- and 5-year-old preschoolers either as a native or a foreign individual. Children were then given the opportunity to help or cooperate with her across five playful tasks. To probe the generalizability of our observations [36], we tested children in two countries, France and Hungary.

Our first hypothesis was that preschoolers in both countries would be more reluctant to help or cooperate with a foreign (vs. a native) adult and would require more explicit cues before doing so. Since no studies to date have looked at the effect of linguistic group membership on preschoolers’ helpfulness and cooperation, we based this assumption on the study by Kinzler et al. [36] showing that 2.5-year-olds (in France and the US) prefer to allocate a present to a native (vs. a foreign) individual. Similarly, two further studies that looked at imitation, another form of social engagement (although strictly speaking not a form of prosociality), showed that 3-year-olds imitate a native adult more readily than a foreign one [44–46].

Our second hypothesis was that 5-year-olds would be more selective than 4-year-olds and would help or cooperate with the foreign (vs. the native) adult less readily, as suggested by studies with younger children showing that with age, especially between 4 and 5 years, preschoolers become increasingly selective in their prosocial behaviors [2, 28, 34].

Given the evidence suggesting that helping emerges earlier in development than cooperation [21, 48] and that in adults, instrumental helping is guided by automatic or intuitive processes whereas cooperation often requires more controlled or deliberate processes [49], our third aim was to compare children’s responses in the two different task types. We wished to explore whether the language spoken by the experimenter has different effects on helping and cooperation.

## Method

### Participants

Our sample consisted of 85 preschoolers recruited from two public preschools in Paris (N = 43) and Budapest (N = 42). We tested children in two different countries to control for any possible effect of local culture. Participants’ distribution across the experimental conditions with mean ages is reported in Table 1. The two groups of French and Hungarian children were matched on age (4 year olds: t(41) = 1.87, p = .07; 5 year olds: t(44) = 1.83, p = .08).

**Table 1:** Distribution of participants in the two experimenter language conditions and at the two test locations with mean ages.

	Condition	Age group			
		4yr olds		5yr olds	
		N	mean age	N	mean age
French sample	Native	12 (5 females)	3.92 (age range = 3.6–4.4 years)	10 (4 females)	4.91 (age range = 4.4–5.3 years)
	Foreign	10 (5 females)		11 (6 females)	
Hungarian sample	Native	10 (6 females)	4.11 (age range = 3.7–4.6 years)	11 (6 females)	5.02 (age range = 4.5–5.5)
	Foreign	9 (5 females)		12 (5 females)	

Eighteen additional children (11 French and 7 Hungarian) were recruited, but not included in the study, either because they lived in a bi- or multilingual family (n = 9 in France and n = 6 in Hungary) or because they did not wish to take part (n = 2 in France and n = 1 in Hungary). We decided to exclude bilingual children as they may be familiar with other speakers and therefore their perception of and response to foreign speakers may be different compared to children raised in monolingual families [50]. Written consent to test in the schools was obtained from each school’s principal. We distributed information letters to parents and contacted those who were interested in order to obtain their signed informed consent. The study was approved by the Ethics committees of Paris Nanterre University (Ethics Committee of the Department of Psychology and Educational Sciences, Paris Ouest Nanterre La Défense University, Paris) and Eötvös Loránd University (Ethics Committee of the Faculty of Education and Psychology, Eötvös Loránd University, Budapest). All participating children’s parents gave their signed informed consent.

For each condition and age group children were recruited from the same class and only one class was included per condition and age in each country (four classes took part in France and four in Hungary, two classes with 4-year-olds and two classes with 5-year-olds).

### Procedure

The experiments took place within the schools in a quiet room that was familiar to the child. Testing was conducted by two female experimenters (E1 and E2). The role of E1 was assigned to a female adult who has spoken both languages since early childhood and could take the role of the native or the foreign adult in both locations. We thus ensured that only the language spoken by E1 changed across conditions, not the person.

Testing was preceded by a familiarization session, where E1 and E2 presented themselves to the group of children to be tested that day. Next, E2 accompanied each child to the testing room and provided the following instruction: ‘Here is my friend, she has brought some games with her, it is now your turn to play with her! I will just sit down here and do some writing while you play.’. Then, E2 sat down at a table at the far end of the room, away from E1 and the child but still at a hearing distance, and E1 started to manipulate the experimental materials. After the test, the children were returned to their classroom and joined the ongoing structured class activity, thus they did not have the opportunity to exchange about the testing session or the tasks.

### Experimental conditions

Children were assigned to one of two conditions, according to how E1 had been presented. The conditions were administered in the following way:

1. Native condition: familiarization with E1 as a native adult. E2 first presented herself in the children’s native language (e.g. in France: ‘Bonjour les enfants, je m’appelle XY.’, meaning: ‘Good morning children, my name is XY.’) and then presented E1, explaining that she was her friend, who had accompanied her (‘Voici mon amie ZW, qui m’a accompagnée aujourd’hui.’, meaning: ‘This is my friend ZW who accompanied me today.’). E1 then greeted the children and presented herself in the local language (‘Bonjour les enfants, je m’appelle ZW.’, meaning: ‘Good morning children, my name is ZW.’). Then E2 told the children that they had both come to the school to play and to try out some interesting games with the children (‘Nous sommes venues pour essayer de nouveaux jeux avec vous.’, meaning: ‘We have come to try new games with you.’).
2. Foreign condition: familiarization with E1 as a foreign adult. The procedure was identical to the Native condition with the only difference that when E2 presented E1, she explained that E1 had come from a different country (‘Voici mon amie ZW, qui m’a accompagnée aujourd’hui et qui vient de la Hongrie.’, meaning: ‘This is my friend ZW who accompanied me today and who comes from Hungary.’). E1 then greeted the children and presented herself in the foreign language (in Hungarian: ‘Sziasztok gyerekek, ZW vagyok.’, meaning: ‘Good morning children, my name is ZW.’).

### Experimental tasks and scoring

The following five tasks were proposed to children in a counterbalanced order (five different orders were determined in advance and the experimenters proceeded in a different order with each child). Two were helping tasks (Clothespin and Two Buttons tasks), three were cooperation tasks (Elevator, Trampoline and Tube tasks). Separate analyses were conducted for these two types of tasks as well as for the five tasks taken together. Three tasks (Clothespin, Elevator and Trampoline tasks) were adapted versions of tasks designed by Warneken and Tomasello [5], the two others (Two Buttons and Tube tasks) were designed for this study.

1. Clothespin Task: E1 is clipping pieces of fabric to a clothesline and drops a clothespin out of reach.
2. Two Buttons Task: E1 sits down next to a button that emits a clicking noise when pressed, she listens to the noise and plays a rhythm with the clicking sound. She tries to reach for a second identical button at the other end of the table, but cannot reach it, as it is too far.
3. Elevator Task: E1 sits behind a small table and tries to recover the ball from a container that is embedded in the surface of a small table. A transparent screen separates her from the container. She lifts the container from below and tries to reach from over the top of the screen to retrieve it, but fails because her arm is not long enough.
4. Trampoline Task: E1 holds two corners of a square-shaped cloth and tries to fling a plastic ball with it in the air, but fails because alone she cannot expand the cloth tightly enough for the ball to bounce off its surface.
5. Tube Task: E1 sends a small plastic ball down a tube and tries to catch it at the other end with a plastic recipient, but fails because the tube is too long for her to reach all the way.

**Fig 1:**
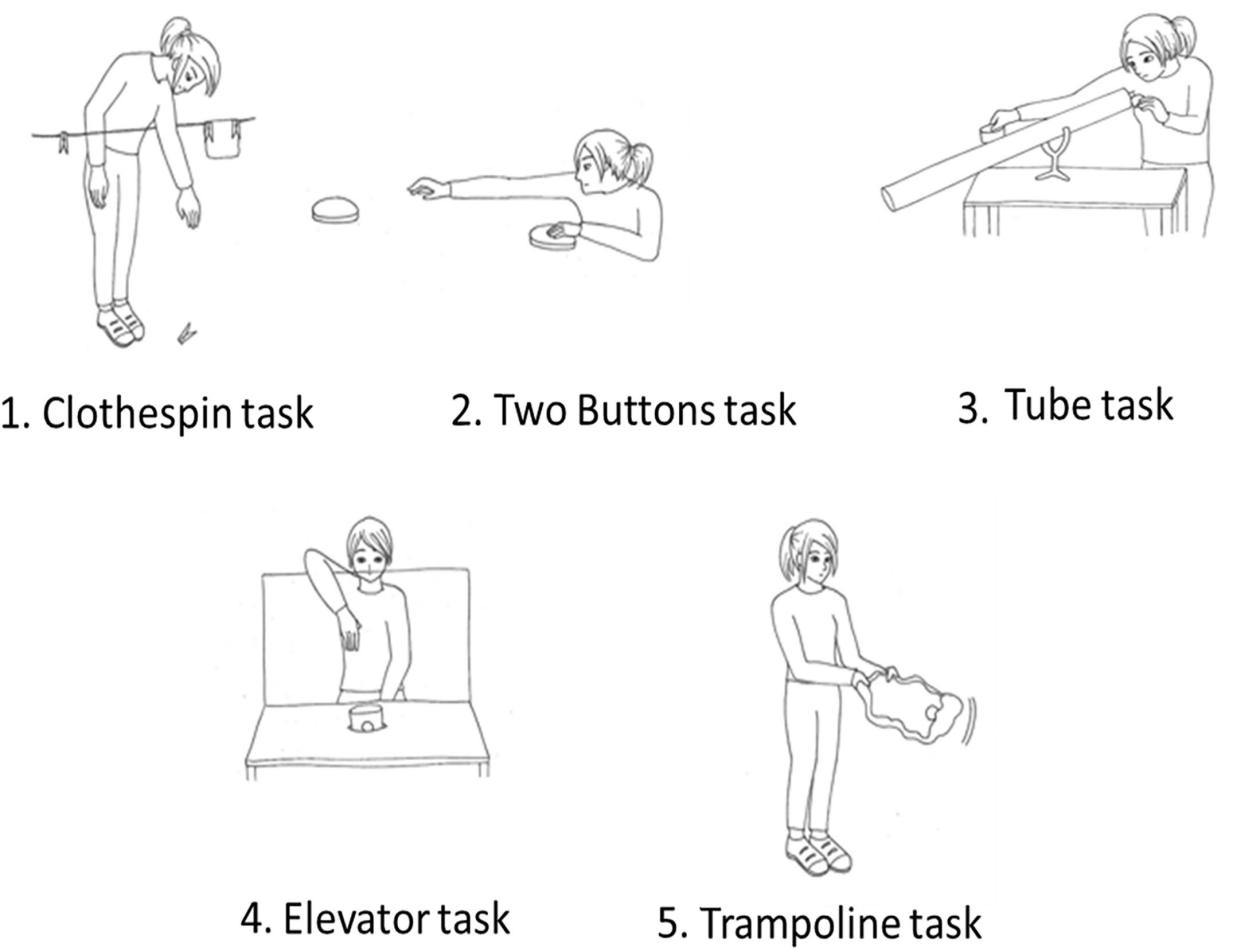
Illustration of the five tasks that were proposed to children to assess their prosocial behaviors. The Clothespin- and the Two Buttons tasks measured helping and the Elevator-, Trampoline- and Tube tasks measured cooperation. Three of these were adapted versions of tasks designed by Warneken and Tomasello [5], the others were designed for the current study.

Fig 1 illustrates the materials used for the five tasks.

To explore how the strength of cues indicating E1’s state modulates children’s responses, during each task, E1 provided progressively more explicit nonverbal cues as to her goal and what could help her. The cues and their order of presentation were the same across tasks. The first cue communicated E1’s general need and frustration (up to three attempts to reach the object, accompanied by nonverbal expression of frustration, without addressing the child, putting hands up, looking around, uttering ‘hmmm’ to herself), without addressing the child. The second cue was a nonverbal request to get the object, by alternating gaze between the object and the child while pointing towards it. Finally, the third cue was an explicit verbal request from E2 to help E1 (‘Can you help her get it?’). To ensure that this protocol was identical across tasks, E1 trained to deliver the protocol in a standardized manner, producing the cues in the same way and with the same duration. Each cue was presented for 5–7 seconds and if the child did not respond within this timeframe, then E1 (or E2 for the third cue) waited for an additional 10 seconds before proceeding to the next cue.

In each task, children’s responses were scored on an ordinal scale, based on whether the child helped or cooperated with E1 or not and on the explicitness of the cues required:

0 = child does not help or cooperate.
1 = helps or cooperates upon explicit verbal request by E2.
2 = helps or cooperates upon nonverbal request by E1.
3 = no request required, helps or cooperates upon expression of E1’s state of need.

This produced a prosociality score (ProsocSc) for each of the five tasks. We conducted a reliability test to assess the internal consistency of our measure. The value of Cronbach’s alpha was 0.57, which we consider acceptable given the limited number of test items and the broad range of behaviors that fall under the tested construct, prosociality. The exclusion of any task did not increase the level of internal consistency obtained, indicating that all five tasks were worthy of retention.

As our main analysis, we performed Generalised Estimating Equations (GEE) analyses to investigate the effects of experimenter language, age and task type on prosociality scores. We chose the GEE method because it is particularly adapted to data clustered per subject (the same children were tested for all five tasks), it can be conducted with an ordinal outcome variable (ProsocSc) and allows multivariate comparisons, probing for eventual interactions between independent variables (Experimenter language, Age and Task type). We also analysed children’s response latency as an implicit measure of the social evaluative mechanisms children rely on when deciding whether to help or cooperate. These results are reported in the Additional materials section.

One quarter (25%) of the sessions (22 children) were independently coded by a second rater who was unaware of the hypotheses of the study. Agreement between the two raters was excellent (ICC = .91).

In 5.5% of tasks, the child did not help or cooperate with E1 even after specific verbal request from E2. In these cases, the response was scored 0 and included in the analyses. We decided to do so based recent data showing that helping behavior in children is independent from general differences in sociability and shyness [21], which suggests that when a child does not produce any responses in a helping task, this is generally not due to any interference of shyness, but can be considered a form of unwillingness to help or cooperate.

To assess the effect of E2’s eventual intervention at the end of tasks where children did not help or cooperate (i.e. the child scored 1 or 0) on children’s responses in the subsequent tasks, we examined the association between the total number of preceding prompts (maximum 4) and ProsocSc in each task. A chi-square test of independence showed no significant association between the two (χ^2^(9) = 10.88, p = .284), indicating that E2’s intervention did not bias children’s subsequent responses.

## Results

Preliminary Mann-Whitney U tests showed no significant effect of test location (child tested in France vs. Hungary: U = 710, p = 0.117) or gender (male vs. female child: U = 845, p = 0.769). For our main analyses we therefore collapsed data from these samples.

**Fig 2:**
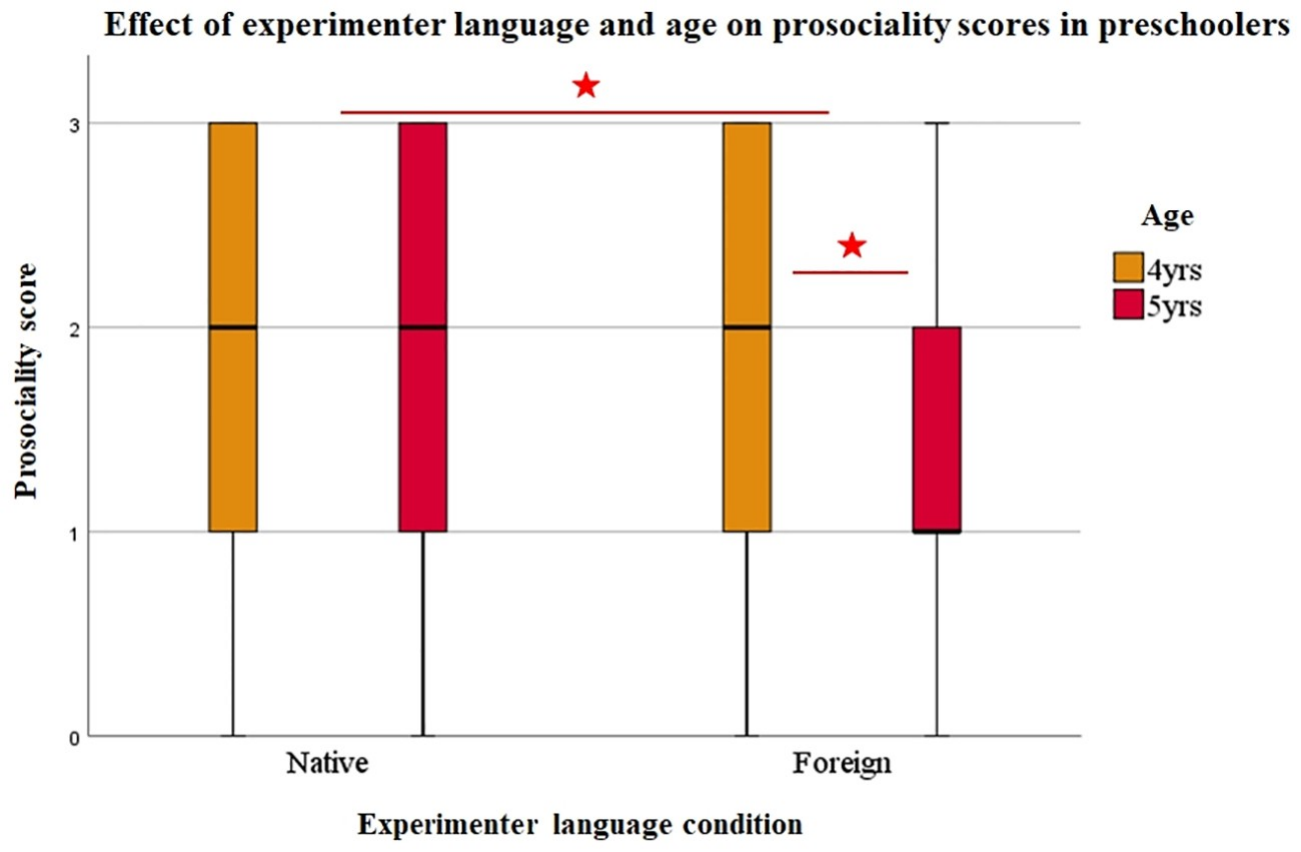
Effect of experimenter language and age on prosociality scores in preschoolers. Experimenter language (Native vs. Foreign) had a significant effect on 4- and 5-year-old preschoolers’ prosociality scores (Wald χ2 = 7.94, df = 1, p = .005), with overall lower scores in the Foreign condition than in the Native condition (U = 16684, p = .001). The interaction between Partner language and Age was also significant (Wald χ2 = 16.10, df = 3, p = .001), as Experimenter language had a significant effect in 5-year-olds but not in 4-year-olds (Wald χ2 = 11.32, df = 1, p = .001; Wald χ2 = 2.05, df = 1, p = .15, respectively). This shows that 5-year-old preschoolers, unlike 4-year-olds, were significantly more reluctant to help or to cooperate when the experimenter was foreign as compared to when she was a native person.

**Fig 3:**
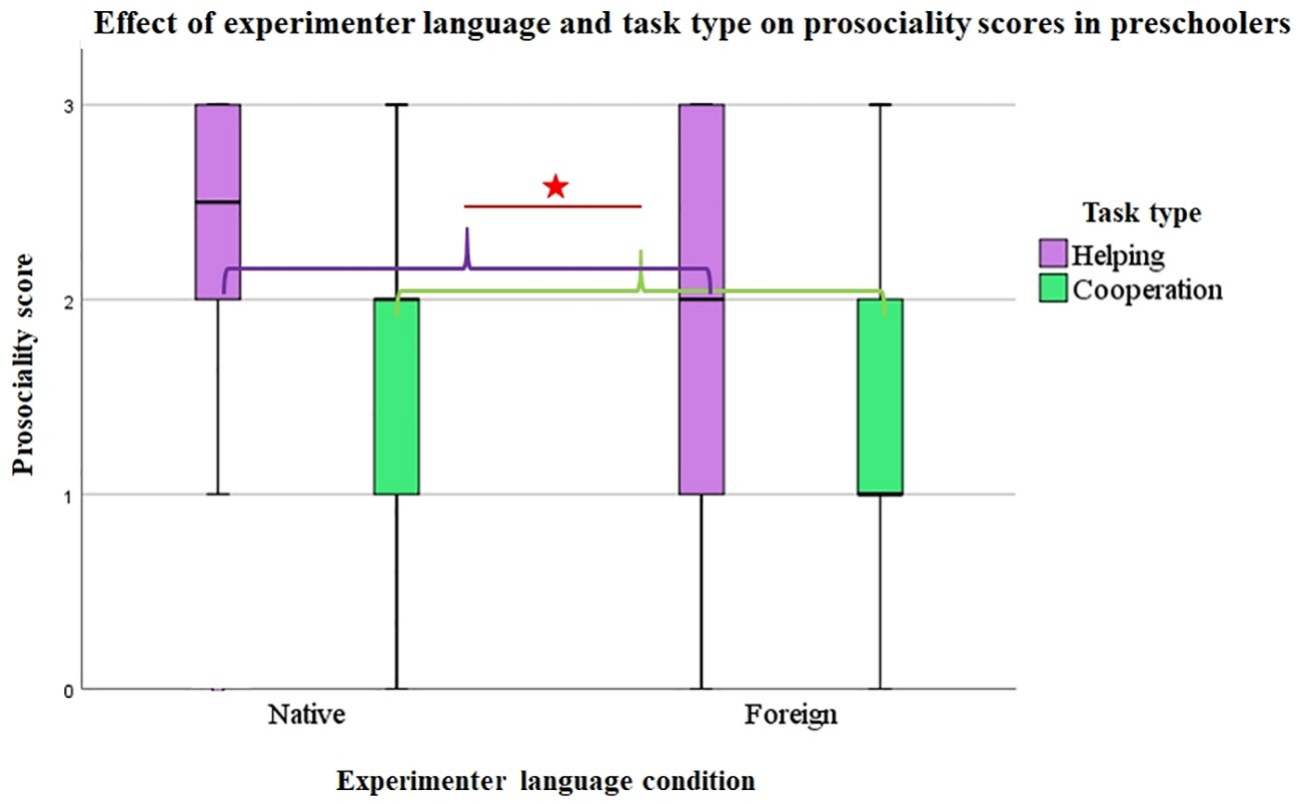
Effect of experimenter language and task type on prosociality scores in preschoolers. Task type (Helping vs. Cooperation) had a significant effect on preschoolers’ prosociality scores (Wald χ2 = 15.44, df = 1, p = .001), with overall lower scores in the Cooperation than in the Helping tasks (U = 14398, p = .001). We found no interaction between Experimenter language and Task type, indicating that Experimenter language had a similar effect for both Task types.

We performed GEE analyses to explore the effects of Experimenter language (Native vs. Foreign), Age (4y vs. 5y) and Task type (Helping vs Cooperating) on prosociality scores (ProsocSc). The results showed that Experimenter language had a significant effect on ProsocScs (Wald χ2 = 7.94, df = 1, p = .005), which were overall significantly lower in the Foreign than in the Native condition (U = 16684, p = .001). Task type also had a significant effect (Wald χ2 = 15.44, df = 1, p = .001), with lower ProsocScs in the Cooperation than in the Helping tasks (U = 14398, p = .001). Age did not have an effect (Wald χ2 = 2.84, df = 1, p = .09), however, we found a significant interaction between Experimenter language and Age (Wald χ2 = 16.10, df = 3, p = .001). We did not find further interactions between our variables. Figs 2 and 3 show the effect of Experimenter language on ProsocScs across Age groups and Task types.

### Effect of experimenter language and age

To explore the effect of Experimenter language according to Age, we performed follow-up GEE analyses on ProsocScs within each Age group.

**Fig 4:**
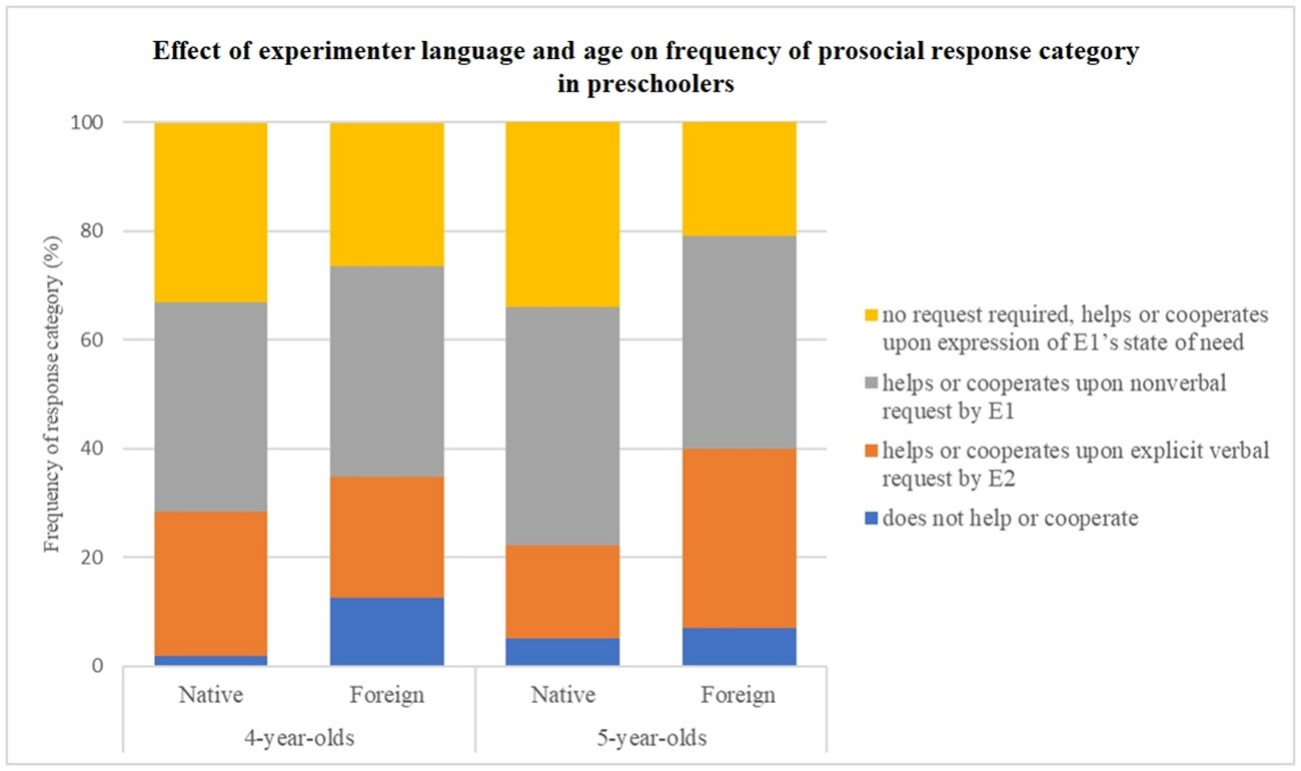
Frequencies of the four prosocial response categories across experimenter language conditions and age groups. Experimenter language had a significant effect in 5-year-olds but not in 4-year-olds (Wald χ2 = 11.32, df = 1, p = .001; Wald χ2 = 2.05, df = 1, p = .15, respectively). Five-year-olds’ responses were categorised as ‘no request required, helps or cooperates upon expression of E1’s state of need’ (score 3) significantly less frequently in the Foreign (21%) than in the Native condition (34%, Wald χ2 = 6.76, df = 1, p = .009). Also, they ‘helped or cooperated upon verbal request by E2’ (score 1) more frequently in the Foreign (33%) than in the Native condition (17%, Wald χ2 = 9.07, df = 1, p = .003). These results indicate that 5-year-olds, but not 4-year-olds, were selective in their prosocial behaviors.

We found that Experimenter language had a significant effect in 5-year-olds but not in 4-year-olds (Wald χ2 = 11.32, df = 1, p = .001; Wald χ2 = 2.05, df = 1, p = .15, respectively). Comparisons of the frequency of each response category in 5-year-olds according to Experimenter language showed that their responses were categorised as ‘no request required, helps or cooperates upon expression of E1’s state of need’ (score 3) significantly less frequently in the Foreign (21%) than in the Native condition (34%, Wald χ2 = 6.76, df = 1, p = .009). Also, they ‘helped or cooperated upon verbal request by E2’ (score 1) more frequently in the Foreign (33%) than in the Native condition (17%, Wald χ2 = 9.07, df = 1, p = .003). Fig 4 shows the frequencies of the four response categories across Experimenter language conditions and Age groups.

These results indicate 5-year-olds, but not 4-year-olds, were selective in their prosocial behaviors. They helped or cooperated with the foreign experimenter significantly less frequently than with the native experimenter and required an explicit verbal prompt by the other experimenter more often to do so.

### Effect of task type

We ran separate follow-up GEE analyses for the Helping and the Cooperation tasks to explore the effect of Experimenter language and Age separately for these two Task types.

In the two Helping tasks, Experimenter language had a significant effect (Wald χ2 = 3.96, df = 1, p = .047), as ProsocScs were overall significantly lower in the Foreign than in the Native condition (U = 2779, p = .003). Age did not have an effect (Wald χ2 = 1.20, df = 1, p = .27) and we did not find an interaction between the two variables either. Comparisons of the frequency of each response category in the Helping tasks according to Experimenter language showed that children ‘helped upon verbal request by E2’ (score 1) more frequently in the Foreign (26%) than in the Native condition (15%, Wald χ2 = 34.35, df = 1, p = .045).

In the three Cooperation tasks, Experimenter language again had a significant effect (Wald χ2 = 12.73, df = 1, p = .001), as ProsocScs were overall significantly lower in the Foreign than in the Native condition (U = 5743, p = .001). Age did not have an effect (Wald χ2 = .65, df = 1, p = .42) and we did not find an interaction between the two variables either. Comparisons of the frequency of each response category showed that children’s responses were categorised as ‘does not cooperate’ (score 0) more frequently in the Foreign (15%) than in the Native condition (2%, Wald χ2 = 9.43, df = 1, p = .002). Children also ‘cooperated upon expression of E1’s state of need’ (score 3) significantly less frequently (11%) in the Foreign than in the Native condition (33%, Wald χ2 = 5.74, df = 1, p = .017).

**Fig 5:**
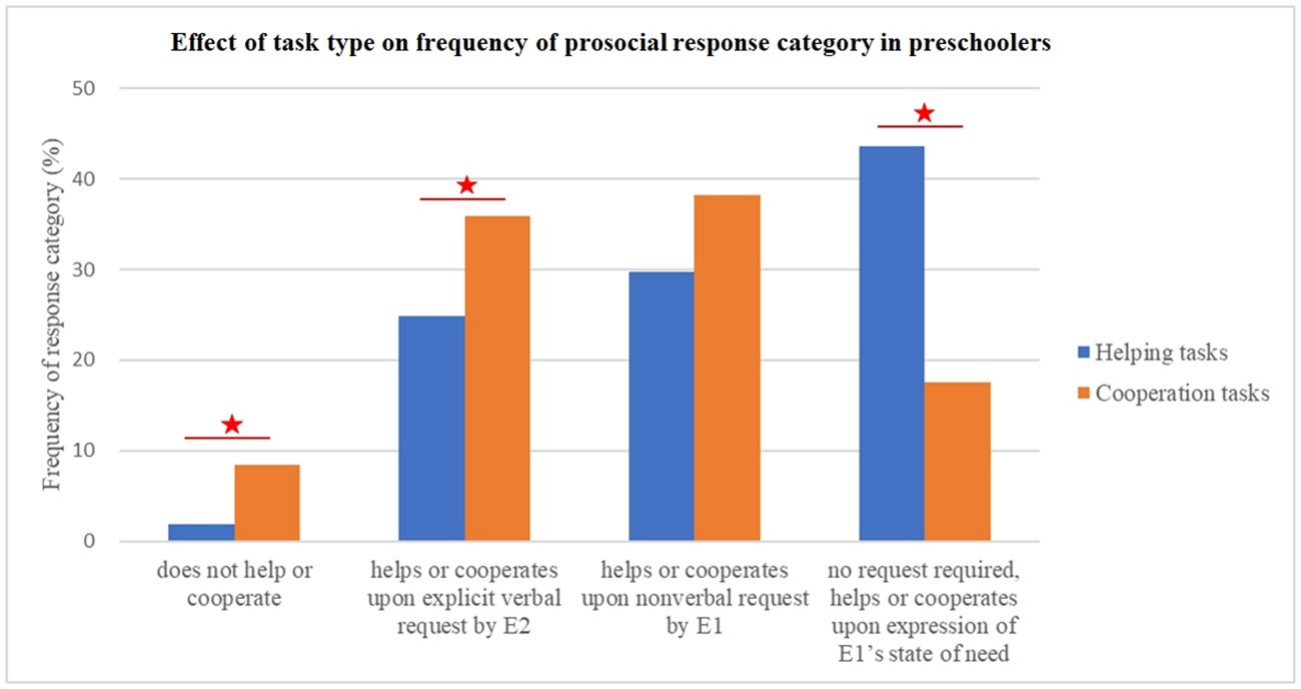
Frequencies of the four prosocial response categories in the two task types. In cooperation tasks, 4- and 5-year-old preschoolers’ responses were significantly more frequently categorised as ‘does not help or cooperate’ (score 0) and ‘requires verbal request by E2’ (score 1) than in helping tasks (p = .004 and p = .02). Children also ‘helped or cooperated upon expression of E1’s state of need’ (score 3) significantly less frequently (p = .001) in cooperation tasks. These results indicate that children were overall more reluctant to respond prosocially in the cooperation tasks than in the helping tasks.

Finally, we compared the frequency of each response category in the two Task types. In the Cooperation tasks, children’s responses were significantly more frequently categorised as ‘does not help or cooperate’ (score 0) and ‘requires verbal request by E2’ (score 1) than in the Helping tasks (score 0: 8% in Cooperation and 2% in Helping tasks, Wald χ2 = 6.46, df = 1, p = .011; score 1: 36% in Cooperation and 25% in Helping tasks, Wald χ2 = 6.27, df = 1, p = .012). Children also ‘helped or cooperated upon expression of E1’s state of need’ (score 3) significantly less frequently in the Cooperation tasks (18%) than in the Helping tasks (44%, Wald χ2 = 33.24, df = 1, p = .001). Fig 5 shows the frequencies of the four response categories in the two Task types.

These results indicate that although experimenter language affected responses significantly for both task types, children were overall more reluctant to respond prosocially in the cooperation tasks than in the helping tasks.

## Discussion

The present study investigated how the language spoken by an adult influences prosocial behaviors, namely helpfulness towards and cooperation with this person in preschoolers, a question that has not been addressed by previous studies. We presented an experimenter to 4- and 5-year-olds either as a foreigner, who did not speak the local language or as a native person. Children were then given the opportunity to help or cooperate with the experimenter across five playful tasks.

Our first hypothesis was that preschoolers would show selectivity in their responses and would be more reluctant to help or cooperate with the foreign experimenter versus the native one. This was partially confirmed as 5-year-olds, but not 4-year-olds, required significantly more cues and prompts regarding the experimenter’s need in order to help or cooperate when she was previously introduced as a foreigner as compared to when she was introduced as a native person. This result is in line with findings of earlier studies showing similar selectivity in young children’s responses when they allocate presents (preferring a native over a foreign recipient, [36]) or when they choose who to imitate or trust (preferring to imitate and to trust linguistic in-group over out-group members, [44–46]). The same pattern of responses was observed in French and Hungarian children, suggesting that our findings are generalizable, at least in Western cultures [51]. We can therefore conclude that language is a natural cue for categorising others and that this categorisation guides children in their actual, real-time decisions about whether to behave prosocially with a potential partner. We know that language also guides children in identifying persons with whom they share knowledge, culture and norms [20, 41, 47] or individuals to learn from or to bond with [52, 53]. It is possible therefore that 5-year-olds in the current study categorised the foreign experimenter as someone with whom they do not share knowledge or norms, someone they cannot learn from or bond with, which in turn led them to be less prosocial with such a person. Whilst these two dimensions, affiliation and trust for learning are interdependent [54] one way to tease them apart in future research could be to gradually introduce subtle linguistic cues (e.g. accent) of the experimenter’s language use, once affiliation is established (e.g. following a playful warm-up phase). It is conceivable that such cues would not influence affiliation towards the experimenter but still inform children about their knowledgeability.

Our second hypothesis was that older (vs. younger) preschoolers would be more selective in their prosocial responses, as suggested by earlier studies [2, 28, 34, 46]. Our results confirmed this hypothesis as 4-year-olds, unlike 5-year-olds, helped and cooperated equally with the foreign and the native experimenter. We can therefore conclude that with age, children’s motivations to act in a prosocial manner become more sophisticated as new means of social evaluation and categorization develop. The causes of this increasing selectivity may differ across age groups and situations, but likely candidate mechanisms discussed in literature range from expectations of reciprocity based on the attribution of prosocial dispositions [13, 55] to a more general motivation to be prosocial toward positively valenced individuals (such as in-group members) in the absence of immediate opportunities for reciprocity and reputational gain [56]. Older children’s intergroup helping is also influenced by their own desire for a positive social identity, which prescribes ingroup loyalty as a moral principle. In a vignette study by Sierksma et al. [57], 10-year-old native Dutch children evaluated hypothetical helping situations in which the ethnicity (Dutch or Turkish) of the helper and of the recipient were systematically varied. Whilst the refusal to help was generally evaluated very negatively, children were more negative when it happened in an intragroup context (two Dutch ingroup members or two Turkish outgroup members) compared with an intergroup context (a Dutch and a Turkish protagonist).

Interestingly, when the experimenter was foreign, 5-year-olds, but not 4-year-olds, helped or cooperated more frequently upon an explicit verbal request made by the other, native experimenter. Thus, the verbal cue was more powerful in the context of helping and cooperating with a foreign (vs. a native) person. This suggests that explicit verbal indications should be included in classroom procedures and interventions to promote prosocial behaviors in preschoolers.

Finally, our third aim was to compare children’s responses in the helping and the cooperation tasks. We did find a difference in responses, as helpfulness was overall more frequent than cooperation, independently of age or experimenter language. When children cooperated, they did so following more cues and requests than in the helping tasks. It has been shown in adults that instrumental helping is guided by automatic or intuitive processes whereas cooperation often requires more controlled or deliberate processes [49]. It is therefore plausible that instrumental helping is less influenced by contextual factors, such as experimenter language in the current study. Cooperation on the other hand may have required the formation of a shared goal based on the other person’s actions and the selection among several possible actions the ones that complement the other’s actions to reach that goal, which processes were possibly more influenced by contextual factors. The fact that children were overall more helpful than cooperative also resonates with earlier findings showing that helping and cooperation follow different developmental pathways, with helping emerging somewhat earlier in development than cooperation [21, 48].

We must note that the current study is limited to two specific types of prosocial behaviors, namely instrumental helping in out-of-reach contexts and cooperation. Thus, our findings need to be extended to the effect of natural cues and social categorization on other forms of prosocial behavior such as providing information, adhering to social norms or helping when the partner is physically uncomfortable [1, 48].

A further limitation is that although all efforts were made to design playful, lifelike situations with familiar experimenters, our standardised tasks may still have created a less natural atmosphere. The standardized design also meant that children had limited interactions with the experimenters. They were exposed to the language spoken by the experimenters only once, when they introduced themselves in the children’s native or the foreign language. Our results therefore cannot be extended to situations where children have more opportunities to familiarize with the other person and their language. Still, evidence shows that children make up their minds about another person’s linguistic group membership relatively fast, based on first impressions [36, 52]. We believe therefore, that our design accurately mirrors everyday situations where children encounter a new person for the first time. An interesting perspective for future research is to explore variability in naturally occurring, spontaneous prosocial behaviors in preschoolers [57].

As children were recruited from the same class for each condition and only one class was included per condition and age in each country, it is conceivable that the group effects we observed could be attributable to confounding factors linked to the particular social or educational dynamics of each class. It is highly unlikely, however, that such factors would bias results in the same direction in two different countries. Also, we have found no examples in literature for potential sources of such systematic bias leading to children in an entire class being overall significantly more or less prosocial than average.

Finally, a general concern regarding studies of children’s prosocial behavior is that children’s helpfulness or cooperation might simply be driven by their motivation to interact with others [58] rather than reflect a genuinely prosocial motivation [16]. A recent study, however, shows that young children’s helping behavior is independent from general differences in sociability and shyness [21], we would therefore argue that possible variations in children’s trait sociability, as well as other differences in personality, could not have driven our results.

This study is the first to have looked at how language as a social marker directly influences helpful and cooperative behaviors in preschoolers, adding to the growing body of literature on the effect of group membership on social and moral behavior [37, 59–62]. Earlier research in this field has focussed on sharing behaviors and the effect of the language on helping and cooperation have not been investigated to date in young children. A further novelty of the present study is to have observed children’s actual helpful and cooperative behaviors, rather than relying on indirect measures of prosociality, such as different forms of reasoning about the partner [34, 37, 58, 63].

Studies show that the linguistic in-group bias observed in our study can be modulated by additional factors such as the knowledgeability or the moral behavior of the other person. Thus, even though preschoolers prefer native speakers compared to non-native speakers, they override this preference when the native speakers prove inaccurate in labeling familiar words [45] or when they see a native-accented speaker describe antisocial actions he or she committed and the non-native accented speaker describe prosocial actions he or she performed [52]. Cues also seem to be organized hierarchically, with language emerging as a stronger cue than race at 5 years of age [43, 64, 65], indicating that children are sensitive to cultural cues beyond physical similarities.

We believe that investigating the direct effect of natural cues on preschoolers’ actual behavior is an important emerging avenue for research in a changing society where preschoolers increasingly participate in multilingual groups. An important question for future research is to investigate how interventions, such as techniques that encourage children to practice perspective taking [66] or to imagine how a recipient of help feels [67] promote prosocial behaviors in classrooms.

### Additional materials

#### Effect of experimenter language on response latency

In addition to the behavioral scoring reported in the main body of our research article, we also analysed children’s response latency as an implicit measure of the social evaluative mechanisms children rely on when deciding whether to help or cooperate. Specifically, based on adults’ studies showing that helping is modulated by context (e.g.: Rand, 2016), we expected that children make decisions more rapidly when facing an in-group member vs an out-group member.

For each task we calculated the child’s response latency in seconds (measured from the videoframe where E1 first expressed her state of need to the frame where the child touched the target object and averaged these latencies across the five tasks (RL) for each child. The duration of time required for the experimenter to manipulate the objects (e.g.: hanging the cloths, trying to recover the ball from the container) did not count towards response latency, only the time required to progress from one cue to the next- up to the cue where the child eventually responded.

**Fig 6:**
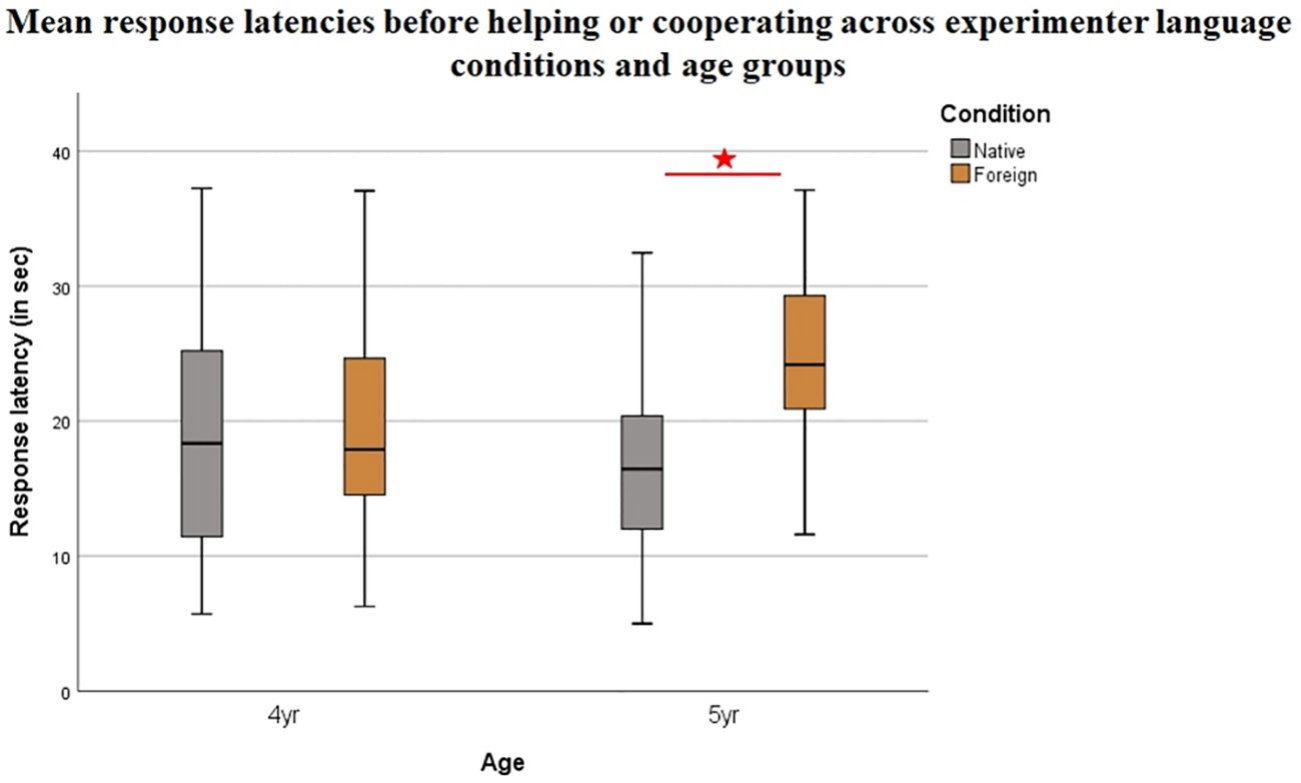
Preschoolers’ mean response latencies across experimenter language conditions and age groups in helping and cooperation tasks combined. Experimenter language had a significant effect on mean response latencies (F(3, 84) = 10.66, p = .002, partial η^2^ = .12). The effect was stronger at 5 years, when preschoolers showed significantly greater response times in the Foreign condition (M_RLForeign5y_ = 25.14, SD_RLForeign5y_ = 6.53) vs. the Native condition (M_RLNative5y_ = 15.94, SD_RLNative5y_ = 6.29, p = .004).

An ANOVA (N = 85) revealed a significant main effect of Experimenter language on RL (*F*(3, 84) = 10.66, p = .002, partial η2 = .12). Preschoolers were significantly slower to respond in the Foreign (M_RLForeign_ = 22.55, SD_RLForeign_ = 7.86) than in the Native condition (M_RLNative_ = 17.1, SD_RLNative_ = 7.24, p = .01). We found no significant main effect of Age (*F*(3, 84) = 1.18, p > .05), however, the interaction between Experimenter language and Age was significant (*F*(3, 84) = 6.33, p = .01, partial η2 = .07). The effect was stronger at 5 years, when preschoolers showed significantly greater response latencies before providing help in the Foreign condition (M_RLForeign5y_ = 25.14, SD_RLForeign5y_ = 6.53) vs. the Native condition (M_RLNative5y_ = 15.94, SD _RLNative5y_ = 6.29, p = .004). At 4 years, however, there was no significant difference between the two conditions (M_RLForeign4y_ = 19.41, SD_RLForeignHu4y_ = 8.35, M_RLNative4y_ = 18.22, SD_RLNative4y_ = 8.04, p > .05). Fig 6 shows preschoolers’ mean response latency across experimenter language conditions and age groups.

These results show that 5-year-olds, but not 4-year-olds responded with more delay in the helping and cooperation tasks when the experimenter was foreign than when she was a native person. Four-year-olds did not show such selectivity.

## Supporting information

S1 File (XLSX)

## References

[1] Dunfield KA, Kuhlmeier VA. Classifying prosocial behavior: Children’s responses to instrumental need, emotional distress, and material desire. Child Dev. 2013; 10.1111/cdev.12075 23461793

[2] Warneken F, Tomasello M. The roots of human altruism. Br J Psychol. 2009; 10.1348/000712608X379061 19063815

[3] Eisenberg N. Altruistic Emotion, Cognition, and Behavior (PLE: Emotion). Altruistic Emotion, Cognition, and Behavior (PLE: Emotion). 2014 10.4324/9781315746135

[4] Yarrow MR, Waxler CZ, Barrett D, Darby J, King R, Pickett M, et al Dimensions and Correlates of Prosocial Behavior in Young Children. Child Dev. 1976; 10.2307/1128290

[5] Warneken F, Tomasello M. Helping and cooperation at 14 months of age. Infancy. 2007; 10.1111/j.1532-7078.2007.tb00227.x 33412734

[6] Brownell CA, Svetlova M, Nichols S. To share or not to share: When do toddlers respond to another’s needs? Infancy. 2009/01/01. 2009;14: 117–130. 10.1080/15250000802569868 22639549 PMC3359011

[7] Roth-Hanania R, Davidov M, Zahn-Waxler C. Empathy development from 8 to 16 months: Early signs of concern for others. Infant Behav Dev. 2011; 10.1016/j.infbeh.2011.04.007 21600660

[8] Liszkowski U, Carpenter M, Striano T, Tomasello M. 12- and 18-month-olds point to provide information for others. J Cogn Dev. 2006; 10.1207/s15327647jcd0702_2

[9] Warneken F, Chen F, Tomasello M. Cooperative activities in young children and chimpanzees. Child Dev. 2006; 10.1111/j.1467-8624.2006.00895.x 16686793

[10] Rheingold HL, Hay DF, West MJ. Sharing in the Second Year of Life. Child Dev. 1976; 10.2307/1128454

[11] Rheingold HL. Little Children’s Participation in the Work of Adults, a Nascent Prosocial Behavior. Child Dev. 1982; 10.2307/1129643

[12] Sebastián-Enesco C, Hernández-Lloreda MV, Colmenares F. Two and a half-year-old children are prosocial even when their partners are not. J Exp Child Psychol. 2013; 10.1016/j.jecp.2013.05.007 23800679

[13] Warneken F, Tomasello M. The emergence of contingent reciprocity in young children. J Exp Child Psychol. 2013; 10.1016/j.jecp.2013.06.002 23917162

[14] Warneken F, Tomasello M. Extrinsic Rewards Undermine Altruistic Tendencies in 20-Month-Olds. Dev Psychol. 2008; 10.1037/a0013860 18999339

[15] Warneken F, Tomasello M. Parental Presence and Encouragement Do Not Influence Helping in Young Children. Infancy. 2013; 10.1111/j.1532-7078.2012.00120.x

[16] Hepach R, Vaish A, Tomasello M. Young Children Are Intrinsically Motivated to See Others Helped. Psychol Sci. 2012; 10.1177/0956797612440571 22851443

[17] Wynn K. Constraints on natural altruism. Br J Psychol. 2009; 10.1348/000712609X441312 19450383

[18] Hamlin JK, Wynn K. Young infants prefer prosocial to antisocial others. Cogn Dev. 2011; 10.1016/j.cogdev.2010.09.001 21499550 PMC3076932

[19] Quinn PC, Yahr J, Kuhn A, Slater AM, Pascalis O. Representation of the gender of human faces by infants: A preference for female. Perception. 2002; 10.1068/p3331 12375875

[20] Esseily R, Somogyi E, Guellai B. The relative importance of language in guiding social preferences through development. Frontiers in Psychology. 2016 10.3389/fpsyg.2016.01645 27812345 PMC5072223

[21] Grossmann T, Missana M, Vaish A. Helping, fast and slow: Exploring intuitive cooperation in early ontogeny. Cognition. 2020; 10.1016/j.cognition.2019.104144 31765923

[22] Moore C. Fairness in children’s resource allocation depends on the recipient. Psychol Sci. 2009; 10.1111/j.1467-9280.2009.02378.x 19515118

[23] Fehr E, Bernhard H, Rockenbach B. Egalitarianism in young children. Nature. 2008; 10.1038/nature07155 18756249

[24] Paulus M, Moore C. The development of recipient-dependent sharing behavior and sharing expectations in preschool children. Dev Psychol. 2014; 10.1037/a0034169 23978297

[25] Eisenberg N, Miller PA. The Relation of Empathy to Prosocial and Related Behaviors. Psychological Bulletin. 1987; 10.1037/0033-2909.101.1.91 3562705

[26] Vaish A, Carpenter M, Tomasello M. Sympathy Through Affective Perspective Taking and Its Relation to Prosocial Behavior in Toddlers. Dev Psychol. 2009; 10.1037/a0014322 19271837

[27] Over H, Carpenter M. Eighteen-month-old infants show increased helping following priming with affiliation: Research report. Psychol Sci. 2009; 10.1111/j.1467-9280.2009.02419.x 19674388

[28] Olson KR, Spelke ES. Foundations of cooperation in young children. Cognition. 2008; 10.1016/j.cognition.2007.12.003 18226808 PMC2481508

[29] Engelmann J, Haux L, Herrmann E. Helping in young children and chimpanzees shows partiality towards friends. Evolution and human behavior. 2019; 10.1016/j.evolhumbehav.2019.01.003

[30] Carpenter M, Uebel J, Tomasello M. Being mimicked increases prosocial behavior in 18-month-old infants. Child Dev. 2013; 10.1111/cdev.12083 23488734

[31] Meltzoff AN. Foundations for developing a concept of self: The role of imitation in relating self to other and the value of social mirroring, social modeling, and self practice in infancy The self in transition: Infancy to childhood. Chicago, IL, US: University of Chicago Press; 1990 pp. 139–164.

[32] Dunfield KA, Kuhlmeier VA. Intention-mediated selective helping in infancy. Psychol Sci. 2010; 10.1177/0956797610364119 20424094

[33] Vaish A, Carpenter M, Tomasello M. Young Children Selectively Avoid Helping People With Harmful Intentions. Child Dev. 2010; 10.1111/j.1467-8624.2010.01500.x 21077854

[34] Plötner M, Over H, Carpenter M, Tomasello M. The effects of collaboration and minimal-group membership on children’s prosocial behavior, liking, affiliation, and trust. J Exp Child Psychol. 2015; 10.1016/j.jecp.2015.05.008 26112747

[35] Renno MP, Shutts K. Children’s social category-based giving and its correlates: Expectations and preferences. Dev Psychol. 2015; 10.1037/a0038819 25706588

[36] Kinzler KD, Dupoux E, Spelke ES. “Native” Objects and Collaborators: Infants’ Object Choices and Acts of Giving Reflect Favor for Native Over Foreign Speakers. J Cogn Dev. 2012; 10.1080/15248372.2011.567200 23105918 PMC3478775

[37] Dunham Y, Baron AS, Carey S. Consequences of “Minimal” Group Affiliations in Children. Child Dev. 2011; 10.1111/j.1467-8624.2011.01577.x 21413937 PMC3513287

[38] Katz P, Katz I, Cohen S. White children’s attitudes toward Blacks and the physically handicapped: A developmental study. J Educ Psychol. 1976; 10.1037/0022-0663.68.1.20

[39] Bigler RS, Jones LC, Lobliner DB. Social categorization and the formation of intergroup attitudes in children. Child Dev. 1997; 10.2307/1131676 9249964

[40] Coulon M, Guellai B, Streri A. Recognition of unfamiliar talking faces at birth. Int J Behav Dev. 2011; 10.1177/0165025410396765

[41] Kinzler KD, Dupoux E, Spelke ES. The native language of social cognition. Proc Natl Acad Sci. 2007; 10.1073/pnas.0705345104 17640881 PMC1941511

[42] Soley G, Sebastián-Gallés N. Infants Prefer Tunes Previously Introduced by Speakers of Their Native Language. Child Dev. 2015; 10.1111/cdev.12408 26300428

[43] Shutts K, Kinzler KD, McKee CB, Spelke ES. Social Information Guides Infants’ Selection of Foods. J Cogn Dev. 2009; 10.1080/15248370902966636 19809590 PMC2756712

[44] Buttelmann D, Zmyj N, Daum M, Carpenter M. Selective Imitation of In-Group Over Out-Group Members in 14-Month-Old Infants. Child Dev. 2013; 10.1111/j.1467-8624.2012.01860.x 23006251

[45] Kinzler KD, Corriveau KH, Harris PL. Children’s selective trust in native-accented speakers. Dev Sci. 2011; 10.1111/j.1467-7687.2010.00965.x 21159092

[46] Howard LH, Henderson AME, Carrazza C, Woodward AL. Infants’ and Young Children’s Imitation of Linguistic In-Group and Out-Group Informants. Child Dev. 2015; 10.1111/cdev.12299 25263528 PMC4358791

[47] Oláh K, Elekes F, Bródy G, Király I. Social category formation is induced by cues of sharing knowledge in young children. PLoS One. 2014; 10.1371/journal.pone.0101680 25014363 PMC4094464

[48] Svetlova M, Nichols SR, Brownell CA. Toddlers’ Prosocial Behavior: From Instrumental to Empathic to Altruistic Helping. Child Dev. 2010; 10.1111/j.1467-8624.2010.01512.x 21077866 PMC3088085

[49] Zaki J, Mitchell JP. Intuitive Prosociality. Curr Dir Psychol Sci. 2013; 10.1177/0963721413492764

[50] Howard LH, Carrazza C, Woodward AL. Neighborhood linguistic diversity predicts infants’ social learning. Cognition. 2014; 10.1016/j.cognition.2014.08.002 25156630 PMC4225131

[51] Henrich J, Heine SJ, Norenzayan A. The weirdest people in the world? Behav Brain Sci. 2010; 10.1017/S0140525X0999152X 20550733

[52] Kinzler KD, DeJesus JM. Children’s sociolinguistic evaluations of nice foreigners and mean Americans. Dev Psychol. 2013; 10.1037/a0028740 22686180

[53] Okumura Y, Kanakogi Y, Takeuchi S, Itakura S. Twelve-month-old infants show social preferences for native-dialect speakers. Shinrigaku Kenkyu. 2014; 10.4992/jjpsy.85.13024 25272442

[54] Over H, Carpenter M. Putting the social into social learning: explaining both selectivity and fidelity in children’s copying behavior. J Comp Psychol. 2012. 10.1037/a0024555 21767011

[55] Kuhlmeier VA, Dunfield KA, O’Neill AC. Selectivity in early prosocial behavior. Front Psychol. 2014; 10.3389/fpsyg.2014.00836 25120526 PMC4114256

[56] Brosnan SF, De Waal FBM. A proximate perspective on reciprocal altruism. Hum Nat. 2002; 10.1007/s12110-002-1017-2 26192598

[57] Sierksma J, Thijs J, Verkuyten M. Ethnic helping and group identity: A study among majority group children. Soc Dev. 2014; 10.1111/sode.12077

[58] Pletti C, Scheel A, Paulus M. Intrinsic altruism or social motivation-what does pupil dilation tell us about children’s helping behavior? Front Psychol. 2017; 10.3389/fpsyg.2017.02089 29259566 PMC5723414

[59] Abrams D, Van de Vyver J, Pelletier J, Cameron L. Children’s prosocial behavioral intentions towards outgroup members. Br J Dev Psychol. 2015; 10.1111/bjdp.12085 25773274

[60] Engelmann JM, Over H, Herrmann E, Tomasello M. Young children care more about their reputation with ingroup members and potential reciprocators. Dev Sci. 2013; 10.1111/desc.12086 24118719

[61] Hetherington C, Hendrickson C, Koenig M. Reducing an in-group bias in preschool children: The impact of moral behavior. Dev Sci. 2014; 10.1111/desc.12192 24836151

[62] Schug MG, Shusterman A, Barth H, Patalano AL. Minimal-group membership influences children’s responses to novel experience with group members. Dev Sci. 2013; 10.1111/j.1467-7687.2012.01193.x 23278926

[63] Bergin CAC, Bergin DA, French E. Preschoolers’ prosocial repertoires: Parents’ perspectives. Early Child Res Q. 1995; 10.1016/0885-2006(95)90027-6

[64] Kinzler KD, Dautel JB. Children’s essentialist reasoning about language and race. Dev Sci. 2012; 10.1111/j.1467-7687.2011.01101.x 22251299

[65] Kinzler KD, Shutts K, Correll J. Priorities in social categories. Eur J Soc Psychol. 2010.

[66] Weltzien S, Marsh LE, Hood B. Thinking of me: Self-focus reduces sharing and helping in seven- to eight-year-olds. PLoS One. 2018; 10.1371/journal.pone.0189752 29320506 PMC5761840

[67] Sierksma J, Thijs J, Verkuyten M. In-group bias in children’s intention to help can be overpowered by inducing empathy. Br J Dev Psychol. 2014; 10.1111/bjdp.12065 25252035

